# The cardio–pancreatic axis in heart failure: from conceptual framework to empirical evidence

**DOI:** 10.1093/eschf/xvag088

**Published:** 2026-03-23

**Authors:** Olivier C Dams, Marlene A T Vijver, Robert C Verdonk, Dirk J van Veldhuisen

**Affiliations:** Department of Cardiology, University of Groningen, University Medical Centre Groningen, Hanzeplein 1, 9713 GZ Groningen, The Netherlands; Department of Cardiology, University of Groningen, University Medical Centre Groningen, Hanzeplein 1, 9713 GZ Groningen, The Netherlands; Department of Gastroenterology and Hepatology, St.Antonius Hospital, Nieuwegein, The Netherlands; Department of Cardiology, University of Groningen, University Medical Centre Groningen, Hanzeplein 1, 9713 GZ Groningen, The Netherlands

**Keywords:** Heart failure, Pancreas, Exocrine insufficiency, FE-1, Portal hypertension

We read with great interest the recent article by Tun *et al*. titled ‘HFpEF Beyond the Heart: Exploring the Heart–Liver–Pancreas Axis.’ The authors provide a compelling conceptual framework for a cardio–pancreatic axis in heart failure with preserved ejection fraction (HFpEF), identifying venous congestion, impaired perfusion, and systemic inflammation as key drivers of pancreatic (exocrine) dysfunction.^1^ We wish to reinforce this axis by highlighting converging functional, biochemical, and structural evidence that suggests this interaction may represent not only a plausible hypothesis but a reproducible clinical entity across the heart failure (HF) spectrum.

First, the authors cite a high prevalence of pancreatic exocrine insufficiency in hospitalized patients (up to 57%). While this reflects the acute haemodynamic insult of decompensation, this axis also appears to remain active even in stable disease. In a prospective study of a stable, heart failure cohort with reduced ejection fraction, exocrine insufficiency was present in a large proportion of NYHA III/IV patients.^2^ Crucially, this finding was associated with appetite loss and could be attenuated with pancreas enzyme replacement therapy (PERT). These findings indicate that pancreatic dysfunction is not merely a transient phenomenon of acute decompensation but may represent a sustained component of the heart failure syndrome, potentially contributing to progressive metabolic deterioration and cardiac cachexia. This observation is particularly relevant in HFpEF, where metabolic frailty is a major determinant of outcome.

Second, the pancreas appears acutely vulnerable to haemodynamic stress. The authors hypothesize that acute heart failure (AHF) may lead to a paroxysmal loss of function. This is supported by our work demonstrating that AHF is accompanied by significant elevations in serum pancreatic enzymes (lipase and amylase), correlating closely with the severity of congestion as defined by NT-proBNP levels.^3^ These findings indicate that the pancreas acts as an early haemodynamic sensor within the splanchnic circulation. Although direct longitudinal structural data are lacking, we propose that these recurrent paroxysmal insults may accumulate over time, serving as the mechanistic bridge to the sustained structural remodelling. This concept is supported by the inflammatory infiltration and peri-pancreatic haemorrhage documented in post-mortem histopathological analyses in patients with HF.^4^

While much of the existing data originates from mixed HF cohorts, the specific haemodynamic profile of HFpEF, characterized by chronically elevated filling pressures and splanchnic congestion, may represent the ‘perfect storm’ for pancreatic injury. The central role attributed to congestion in the authors’ framework is compelling; however, direct haemodynamic validation remains a critical next step. Insights from other congestive disease states may further support this concept. In liver cirrhosis, portal hypertension and subsequent pancreatic congestion have been associated with pancreatic exocrine insufficiency and fibrotic pancreatic remodelling,^5^ suggesting that sustained venous congestion may adversely affect pancreatic structure and function. A similar mechanism may therefore contribute to pancreatic dysfunction in HF. Future studies incorporating invasive portal and central venous pressure measurements in relation to markers of pancreatic function will be essential to clarify the haemodynamic drivers of pancreatic dysfunction and the role of splanchnic congestion in HF.

Furthermore, the reliance on FE-1 as the current diagnostic cornerstone for EPI presents challenges; its accuracy is often limited in mild insufficiency or confounded by the altered bowel habits common in HF. Importantly, intestinal oedema secondary to venous congestion may dilute stool elastase concentrations and reduce diagnostic specificity, thereby confounding interpretation in HF specifically. To fully realize the clinical potential of this axis, future research should incorporate additional functional markers such as endoscopic pancreatic function tests or consider pragmatic trials of PERT in patients exhibiting the ‘cardio-pancreatic’ cachectic phenotype.

In conclusion, the integration of functional, biochemical, and structural data suggests that the pancreas should be recognized as a clinically relevant target organ in the systemic syndrome of heart failure. We commend the authors for their insightful review and advocate for the inclusion of pancreatic assessment and invasive portal haemodynamic validation in future HF(pEF) research protocols.

Sincerely,

Olivier C. Dams, cardiologist in-training University Medical Centre Groningen

## Declarations

### Data Availability

No data were generated or analysed for this manuscript.
